# Otolith shape variability and associated body growth differences in giant grenadier, *Albatrossia pectoralis*

**DOI:** 10.1371/journal.pone.0180020

**Published:** 2017-06-28

**Authors:** Cara J. Rodgveller, Charles E. Hutchinson, Jeremy P. Harris, Scott C. Vulstek, Charles M. Guthrie

**Affiliations:** 1 Auke Bay Laboratories, Alaska Fisheries Science Center, National Oceanic and Atmospheric Administration, National Marine Fisheries Service, Juneau, Alaska, United States of America; 2 Resource Ecology and Fisheries Management Division, Alaska Fisheries Science Center, National Oceanic and Atmospheric Administration, National Marine Fisheries Service, Seattle, Washington, United States of America; Department of Agriculture and Water Resources, AUSTRALIA

## Abstract

Fish stocks can be defined by differences in their distribution, life history, and genetics. Managing fish based on stock structure is integral to successful management of a species because fishing may affect stocks disproportionately. Genetic and environmental differences can affect the shape and growth of otoliths and these differences may be indicative of stock structure. To investigate the potential for speciation or stock structure in giant grenadier, *Albatrossia pectoralis*, we quantified the shape of female giant grenadier otoliths and compared body growth rates for fish with three otolith shapes; shape types were classified visually by an experienced giant grenadier age reader, and were not defined by known distribution or life history differences. We found extreme variation in otolith shape among individuals; however, the shapes were a gradation and not clearly defined into three groups. The two more extreme shapes, visually defined as “hatchet” and “comb”, were discernable based on principal component analyses of elliptical Fourier descriptors, and the “mixed” shape overlapped both of the extreme shapes. Fish with hatchet-shaped otoliths grew faster than fish with comb-shaped otoliths. A genetic test (cytochrome *c* oxidase 1 used by the Fish Barcode of Life Initiative) showed almost no variability among samples, indicating that the samples were all from one species. The lack of young specimens makes it difficult to link otolith shape and growth difference to life history. In addition, shape could not be correlated with adult movement patterns because giant grenadiers experience 100% mortality after capture and, therefore, cannot be tagged and released. Despite these limitations, the link between body growth and otolith shape indicates measurable differences that deserve more study.

## Introduction

Fish stocks, identified for management purposes, are defined as being large enough to be self-sustaining and can be differentiated by their life histories [1]. An understanding of stock structure is integral to management when the productivity or population trends of stocks are not congruent [2]. Stocks can be defined by a variety of complementary methods including morphology, genetics, movement patterns, maturity, growth, and other life history characteristics (reviewed in [2]). Otolith shape morphology has been used extensively to aid in stock identification. Stock-specific shapes have been linked to disparate environmental conditions due to migrations to different feeding grounds or spawning areas [3–14], genetic differences [15], or differences in body condition and growth [3, 4, 6, 9, 13, 16]. In the literature, shape differences can be small enough that they cannot be detected without the aid of morphological analysis [3, 4, 12, 13, 14, 16, 17, 18].

Grenadiers (family Macrouridae) are deep-sea fishes related to hakes and cods that occur globally in all oceans. Giant grenadier, *Albatrossia pectoralis*, have a wide geographic distribution, which extends from Baja California, Mexico around the arc of the North Pacific Ocean to Japan [19]. In Alaska, they are abundant in depths >400 m and are caught incidentally in other directed fisheries (total catch is estimated to be ~12,000 to 21,000 mt per year in Alaska) [19]. They have low commercial value, but are considered an important component of the ecosystem because of their large biomass (estimated biomass from 100–1,000 m in Alaska was 1.6 million mt in 2016) [20]. They may be susceptible to overfishing because they are late to mature (age at 50% maturity was estimated to be 23 years old [21]), ~95% of those caught in surveys are female, and all fish die after capture because of the pressure difference experienced when they are brought to the surface [19]. They are managed as two stocks in Alaska: eastern Bering Sea/Aleutian Islands and the Gulf of Alaska [19, 20]. This boundary is used for many federally managed species and is not specific to giant grenadier management.

Giant grenadier have two distinct otolith shapes that have been observed visually, as well as a third shape that appears to be a mixture of the two distinct shapes [21]. Fish with these otolith shapes are not geographically isolated, at least not during the summer months in Alaska; all three shapes have been previously observed in the eastern and central Gulf of Alaska ~1,000 km apart [21]. There is difficulty linking the life history of giant grenadier to otolith shape differences because little is known about their distribution before maturity or their adult movement patterns. Giant grenadier are one of the most abundant species from 400–1,000 m in the North Pacific Ocean [19, 22]. However, very few larvae and juveniles (age 0 to <15 years) have been caught in longline or trawl surveys or fisheries. It is unknown if there are movements associated with spawning because giant grenadier with developed eggs have been found in several locations across the continental slope in the eastern and central Gulf of Alaska during the summer, indicating that there may not be specific spawning areas and because tagging studies are not possible due to mortality post capture [21]. An analysis of otolith shape and associated body growth is an important step in understanding the potential for life history variability and stock structure in this difficult to study species. If there is stock structure and the stocks are not evenly mixed during fishing, there is potential that stocks could be harvested disproportionately to their abundance.

Our objectives were to 1) determine if a set of shape descriptors could be used to classify otoliths into the three shapes observed in previous studies, 2) investigate species-level genetic differences among fish with distinctive otolith shapes, and 3) compare body growth (length at age) of fish with each otolith shape. Diversity in otolith shape and body growth could indicate variation in life history or genetics. A more complete understanding of this diversity could be used to manage fishing pressure on different stocks.

## Methods

### Ethics statement

This work was completed under United States Department of Commerce Scientific Research Permits (SRP 2004–7, SRP 2006–11, SRP 2013–6). These permits allow the capture of multiple fish species managed by the North Pacific Fishery Management Council, including giant grenadier. Collection of biological data in the United States Exclusive Economic Zone by Federal scientists to support fishery research is granted by the Magnuson-Stevens Fishery Conservation and Management Act. Survey operations were conducted away from Steller sea lion closure areas. No species listed as endangered or threatened species were captured.

### Sampling

**Fig 1:**
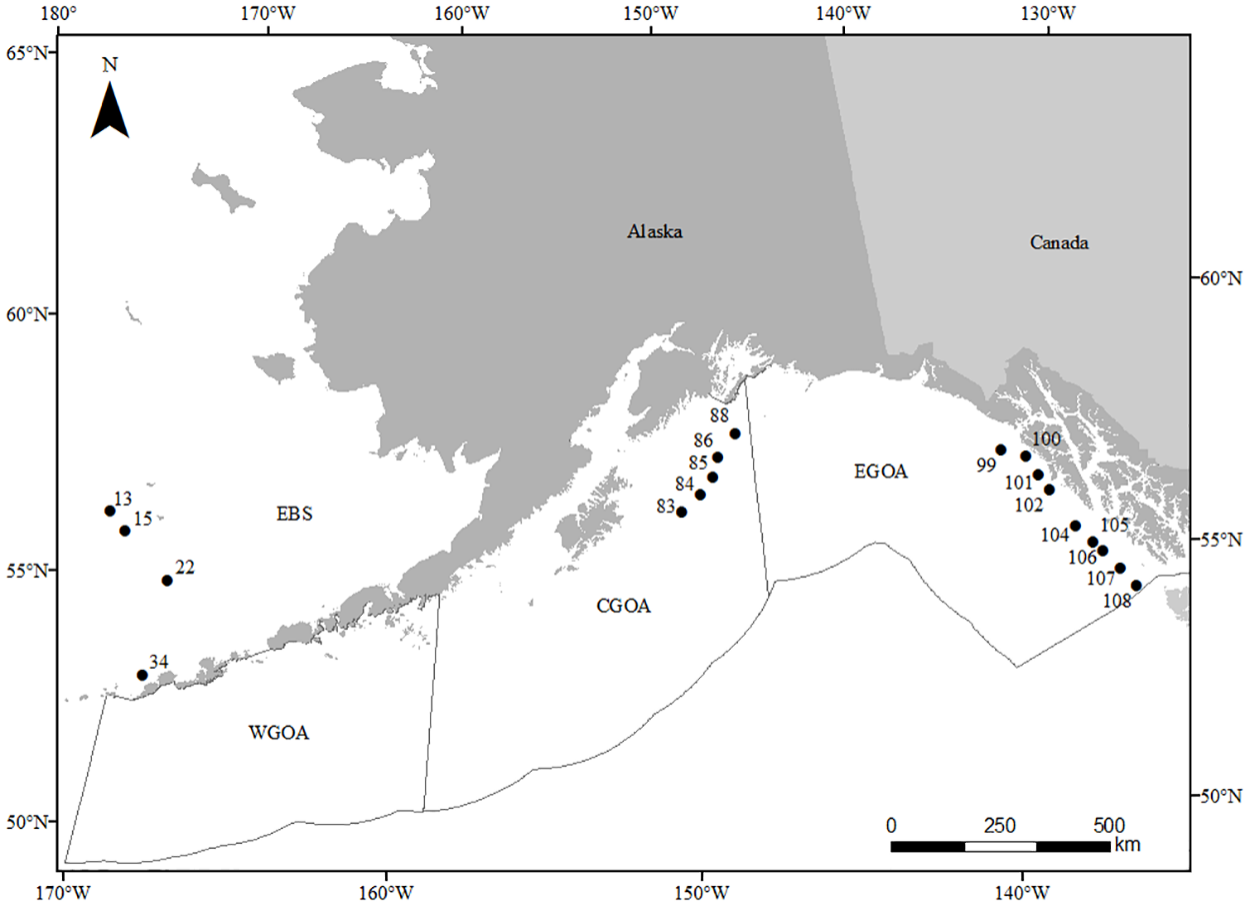
Stations where giant grenadier were sampled in the eastern Gulf of Alaska (EGOA), central Gulf of Alaska (CGOA), and the eastern Bering Sea (EBS).

Female giant grenadier were sampled during the summers (June-July) of 2004, 2006, and 2013 during the Alaska Fisheries Science Center’s annual groundfish longline survey, which samples the continental slope of the Gulf of Alaska, eastern Bering Sea, and eastern Aleutian Islands [23]. For this study samples were collected only in the eastern Gulf of Alaska, Central Gulf of Alaska, and the eastern Bering Sea (Fig 1, Table 1). Stations were spaced 30–50 km apart and sampled from 150–1,000 m. In 2004 and 2006, females were chosen at random in the eastern Gulf of Alaska (2004) and in the central Gulf of Alaska (2006) for a maturity at age study [21]. These samples were used in the current study for the analysis of body growth and not otolith shape.

**Table 1:** Total number (N), average age, and age range (in parenthesis) of giant grenadier by sampling station and year. Samples were either collected in the central Gulf of Alaska (CGOA), eastern Gulf of Alaska (EGOA), and the eastern Bering Sea (EBS). NA indicates that an age range is not applicable because there was one sample.

			Hatchet		Comb		Mixed	
Year	Station	Area	N	Ages	N	Ages	N	Ages
2004	83	CGOA	14	35 (24–49)	14	30 (19–35)	13	37 (25–48)
	84	CGOA	9	30 (24–46)	10	32 (23–43)	9	34 (26–38)
	85	CGOA	8	34 (21–46)	10	32 (24–54)	15	35 (28–40)
	86	CGOA	17	34 (23–45)	5	29 (24–34)	10	35 (29–43)
	88	CGOA	23	30 (21–44)	2	30 (25–35)	8	30 (23–46)
2004 total		CGOA	71	32 (21–49)	41	31 (19–54)	55	36 (23–48)
2006	99	EGOA	1	35 (NA)	0		1	43
	100	EGOA	4	38 (29–47)	4	34 (22–50)	1	42
	101	EGOA	14	32 (24–47)	8	30 (22–37)	7	31 (27–48)
	102	EGOA	10	29 (21–29)	11	29 (16–37)	7	29 (21–35)
	104	EGOA	7	33 (26–45)	13	27 (21–35)	6	27 (15–37)
	105	EGOA	12	35 (27–42)	8	32 (20–58)	5	38 (32–47)
	106	EGOA	11	29 (20–40)	6	28 (17–44)	8	27 (20–34)
	107	EGOA	13	32 (22–49)	0		7	31 (23–49)
	108	EGOA	10	27 (22–32)	5	26 (21–29)	7	30 (21–35)
2006 total		EGOA	82	31 (20–49)	55	29 (16–58)	49	31 (15–49)
2013	13	EBS	20	28 (23–41)	4	33 (22–52)	15	26 (20–37)
	15	EBS	15	28 (23–42)	1	26	18	28 (22–36)
	22	EBS	1	30	0		0	
	34	EBS	8	28 (20–37)	0		0	
	83	CGOA	3	27 (23–31)	0		1	42
	84	CGOA	2	32 (31–33)	3	28 (21–34)	4	32 (26–38)
	85	CGOA	11	31 (27–42)	5	31 (28–34)	4	31 (28–34)
	86	CGOA	7	28 (22–32)	1	26	15	26 (19–32)
	88	CGOA	9	34 (29–45)	5	34 (23–54)	7	36 (22–56)
	107	EGOA	17	33 (23–43)	5	38 (31–45)	17	32 (25–44)
	108	EGOA	19	31 (16–44)	9	30 (20–49)	13	27 (20–42)
2013 total			112	29 (16–45)	33	31 (21–54)	89	29 (19–56)
All years		All	265	30 (16–49)	129	30 (16–58)	193	31 (15–56)

In 2013, samples were collected in the eastern Bering Sea, the eastern Gulf of Alaska, and the central Gulf of Alaska for the purpose of a morphometric analysis of otolith shape (Tables 1 and 2). These samples were also used in conjunction with the 2004 and 2006 samples in the analysis of body growth. Giant grenadier were collected from a depth range of 401–800 m, although they were prevalent in the survey from 400–1,000 m, to minimize any effect that depth may have on otolith shape. Depth range was limited because sample sizes for each otolith shape would not have been substantial enough for a rigorous comparison of shapes by depth. Because fish with all three shapes of the same broad age range were distributed in each geographic area, and because the proportions of each shape were very similar within the Gulf of Alaska, we assume one large population and that samples were independent. Only females were collected to remove any variation in shape associated with sex and because ~95% of the giant grenadier caught on the longline survey are female; males are rarely caught in waters shallower than 1,000 m and have been found in higher numbers in deeper water [24]. For each fish, pre anal fin lengths (PAFL, from the tip of the snout to the start on the anal fin) were measured to the nearest centimeter and mass was measured to the nearest 10 g with a motion-compensating scale. PAFL was used for grenadiers because the tail is very long and fragile and can break off. A small piece of the heart was stored in dimethyl sulfoxide (DMSO) for genetic analyses.

**Table 2:** Number (N), average age, and age range (in parenthesis) of giant grenadier by sampling station and year used in elliptical Fourier shape analyses. Samples were either collected in the eastern Bering Sea (EBS), central Gulf of Alaska (CGOA), or the eastern Gulf of Alaska (EGOA). NA indicates that an age range is not applicable because there was one sample.

			Hatchet		Comb		Mixed	
Year	Station	Area	N	Ages	N	Ages	N	Ages
2013	13	EBS	13	29 (23–35)	4	32 (22–52)	13	26 (20–37)
	15	EBS	15	28 (23–42)	1	26 (NA)	18	28 (22–36)
	22	EBS	1	30 (NA)				
	34	EBS	7	28 (20–37)				
EBS total			36	28 (20–42)	5	30 (22–52)	31	32 (20–45)
	83	CGOA	3	28 (23–31)			1	42 (NA)
	84	CGOA	1	33 (NA)	2	25 (21–28)	4	34 (31–38)
	85	CGOA	10	31 (27–42)	4	30 (28–33)	5	31 (28–34)
	86	CGOA	5	29 (28–32)	1	26 (NA)	11	25 (19–32)
	88	CGOA	5	33 (29–45)	2	27 (23–31)	2	48 (39–56)
CGOA total		CGOA	24	28 (21–33)	9	28 (21–33)	23	31 (19–56)
	107	EGOA	12	33 (23–43)	3	35 (31–39)	13	35 (28–45)
	108	EGOA	15	32 (16–44)	10	30 (20–49)	12	27 (20–42)
EGOA total			27	32 (16–44)	13	31 (20–49)	25	32 (20–45)
Total		All	87	30 (16–45)	27	30 (20–52)	79	29 (19–56)

**Fig 2:**
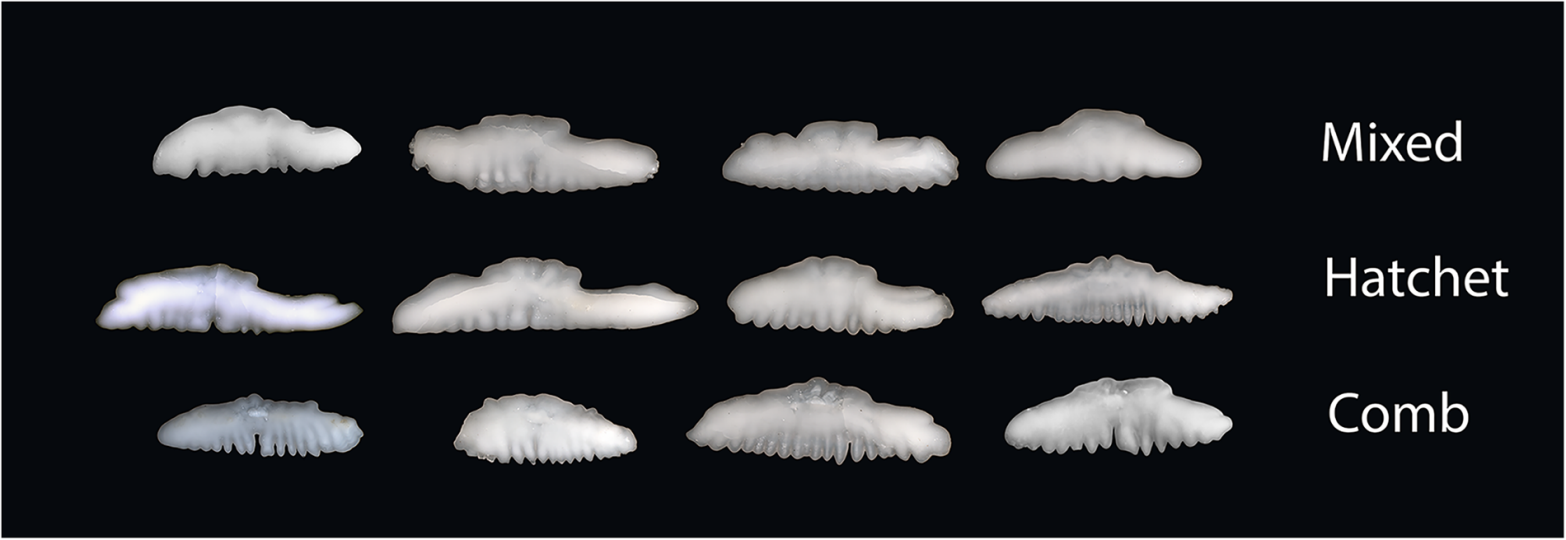
Image of examples of the three otolith shapes in giant grenadier identified visually during age determination.

Unlike in 2004 and 2006, when females were chosen at random, samplers in 2013 were instructed to attempt to collect equal numbers of each of the three otolith shapes, but the official shape was defined later visually by the otolith age reader at the Alaska Fisheries Science Center, who aged all specimens used in this study. Because the samples collected in 2004 and 2006 were collected randomly, they were used for determining the relative proportion of each shape in each sample area, where shape was determined visually by the age reader. In all years, the “hatchet” shape was defined by a narrow posterior end, a fanning out on the anterior end, and a small amount of crenulation on the ventral side (Fig 2). The “comb” shape was rounded on both the anterior and posterior ends that fan out and was deeply crenulated on the ventral side resembling a comb. The “mixed” category was the combination in appearance of the two shapes. Otoltihs were removed for ageing and stored in 50% ethanol solution in 2004 and 2006 and were stored dry in 2013 and rehydrated with glycerin thymol solution.

### Otolith morphometrics

Otoliths were removed from vials and patted dry with a laboratory tissue to remove glycerin thymol. The otoliths were placed under a dissecting microscope at 6.3X magnification with a black background and viewed from their distal side. Whole otolith images were captured using Media Cybernetics Image Pro^®^ 7. Images were also used for an elliptical Fourier analysis of shape, described in the “Elliptical Fourier Analyses” section. Otolith area, perimeter, major axis length, minor axis length, Feret width, and Feret length were collected using Image Pro^®^ 7 measurement tools that were automated with a macro. Feret diameters are similar to measurements using calipers, where an object is measured as the distance between two parallel lines. Positioning the object to yield the largest measurement is termed the Feret length and the position to yield the smallest measurement is the Feret width. These measurements are useful for objects that are irregular or have many indentations, such that a direct measurement through the centroid may not adequately describe the shape. The major axis length is the longest internal distance constrained to pass through the center of mass of the otolith. The minor axis length is the measurement perpendicular to the major axis. Otoliths were weighed to the nearest thousandth of a gram. Morphometric differences among shapes were explored using principal components analyses (PCA) using R [25]. Otoliths were prepared and aged using methods developed for giant grenadier [21]. Some otoliths collected in 2013 were damaged but still ageable; these were not included in the morphometric analysis or the elliptical Fourier analysis (Table 2) but were used in the body growth analysis (Table 1).

A linear model was used to examine significant differences in morphometric measurements among otolith shapes. Measurements of the left otoliths only, for consistency, were used to test for differences using the following general linear model,

$$M_{ij}=\mu +L_{i}+\;A_{i}+\;S_{j}+A_{i}*S_{j}+\;L_{i}*S_{j}+e_{ij},$$ (1)

where *M*_*ij*_ was the morphometric measurement (e.g., major axis length), *μ* is the theoretical population mean, *L*_*i*_ was the length and *A*_*i*_ was the age of the fish, *S*_*j*_ was the categorical otolith shape (three categories), *A*_*i*_ * *S*_*j*_ was the interaction between age and otolith shape, *L*_*i*_ * *S*_*j*_ was the interaction between fish length and otolith shape, and *e*_*ij*_ was the normal, random error. Age and length were included as covariates to account for differences in morphometry associated with fish age or size. Both were included because length and age were not highly correlated (comb shape *R*^2^ = 0.3, *N* = 129; mixed shape *R*^2^ = 0.1, *N* = 193; hatchet shape *R*^2^ = 0.2, *N* = 265). If interaction terms were not significant a reduced model was used without those terms. When there was a significant otolith shape effect for a morphometric measurement, a Tukey-Kramer HSD test was used to test for differences in means between all pairs of otolith shapes [26].

### Elliptical Fourier shape analyses

Otoliths often have a complex shape that lack consistently identifiable points or landmarks and, therefore, may not be sufficiently described by morphometric measurements (such as perimeter, length, and width). Fourier analyses have a number of advantages, including the ability to provide an accurate description of complex or curved shapes [27] and are used for accurate discrimination of stocks or subpopulations. Elliptical Fourier analysis is a group of techniques used to describe curves, like those of an otolith, in terms of cosine waves (also called harmonics) [28]. A series of radii are drawn at equal angles from a centroid to coordinates along the outer edge. Harmonics are fit to these data to describe the contours in the shape. Harmonics are added until at least 99% of the variance in the otolith shape can be reconstructed [29]. In this process a number of ellipses with four Fourier descriptors each are used to describe the shape. Those descriptors can then be examined using a PCA or similar technique [27]. Elliptical Fourier-based techniques have been used successfully to distinguish stocks or species from one another for many taxa worldwide (e.g., [4, 7, 27, 28, 30]). More extensive details of this method are available in the literature [28, 29]. There are several varieties of Fourier analysis (e.g., elliptical Fourier Analysis and fast Fourier transform), but elliptical Fourier analysis was found to describe the grenadier otoliths in this study using the fewest harmonics and Fourier descriptors, which is useful for maximizing statistical power while limiting statistical noise.

Otoliths from the left side of 193 specimens (Table 2) were photographed, each image was converted to a grayscale, and the threshold was adjusted to produce a black-and-white image. From these images, an outline was extracted using the R package “Momocs” (ver. 0.2–6) [31]. The set of outlines were aligned to a common center, oriented to remove discrepancies in positioning, and scaled to centroid size using functions built into the package. Giant grenadier otoliths have strongly irregular, wavy edges that can cause difficulties in fitting the harmonic curves to the shape (Fig 2), requiring a smoothing algorithm to simplify the shapes and to soften the impact of minor variations [32]. Trial runs using 0, 10, 20, and 50 smoothing iterations were conducted, and the number that produced the optimal discrimination of otolith shapes was used for further analysis. After this, an elliptical Fourier analyses was conducted to fit Fourier harmonics to each otolith outline, with subsequent analysis conducted on the set of Fourier descriptors.

Before conducting statistical tests on the Fourier descriptors, they were tested for allometric relationships with either the age of the fish or the Feret length of the otolith using multivariate analyses of variance (MANOVAs). Any significant effect of otolith size or fish age on Fourier descriptors must be removed for an unbiased comparison of shape types [3, 5, 33, 34]. Otolith length was used, and not fish length, because it is less prone to errors [28]. Age was not a significant predictor of the recovered Fourier descriptors (MANOVA, p = 0.154). Otolith length was significantly linked to the overall set of Fourier descriptors (MANOVA, p<0.001).

To determine which of the 45 Fourier descriptors required correction, we examined 45 individual analyses of covariance (ANCOVA) models, one for each descriptor. Each model was used to compare one elliptical Fourier descriptor to Feret length, which was significant in 14 of 45 models. These 14 descriptors were corrected for the effect of otolith length using the pooled-slope, as in [33] using the following formula,

$$FD_{A}=FD_{O}-b\left(L\right),$$ (2)

where *FD*_*A*_ is the adjusted Fourier descriptor, *FD*_*O*_ is the original Fourier descriptor, *b* is the slope, and *L* is otolith length.

After these allometric corrections were made to the Fourier descriptors, a MANOVA was used to test for a significant difference between shape categories based on their harmonic descriptors using the following formula:

$$EF_{ijk}=W_{jk}+\beta_{ik}+e_{ijk}$$ (3)

where *EF*_*ijk*_ is the matrix of the elliptical Fourier descriptor, *k*, for the *j* individual, with shape category *i*, *W*_*jk*_ is the matrix of mean Fourier descriptors for individuals across shape categories, *β*_*ik*_ is the matrix of mean Fourier descriptors values within shape categories, and *e*_*ijk*_ is the matrix of random errors. The MANOVA tests the hypothesis that the set of elliptical Fourier descriptors varies with respect to the otolith shape category, which is the dependent variable. A MANOVA cannot be used to determine the specific shape types that differ. Therefore, a PCA was conducted to help visualize ways the shape of the otoliths differed and whether these results matched the visual categorizations of each otolith into the three shapes.

A linear discriminant analysis was used to develop a function capable of mathematically assigning each otolith to a category based only on its Fourier harmonics. This shape assignment was compared to the visual shape assignment. In this analysis, the “correct” shape was the shape defined by the age reader. Because the same data were used to fit and develop the model, a leave-one-out jack-knife cross-validation method was used to test the effectiveness of the discriminant function.

### Genetic analysis

DNA was extracted from heart tissue samples that had been preserved in DMSO for 341 individuals using QIAGEN DNeasy Blood and Tissue Kits (Qiagen Inc.) to determine if the giant grenadier samples comprised more than one species. An approximately 700bp region of the cytochrome *c* oxidase 1 (COI) mitochondrial gene was amplified using polymerase chain reaction with M13-tailed primer cocktails as described in [35]. The COI gene sequence data are used by the Fish Barcode of Life Initiative (FISH-BOL; www.fishbol.org) as a method for identifying fishes to the taxonomic species level. Resulting amplicons were standardized to a final concentration of approximately 10–15 ng/ul of DNA and were stored at -20°C. Samples were sent to the University of Washington High Throughput Genomics Center for Sanger Sequencing. Mitochondrial sequence data were aligned and processed using CodonCode Aligner (CodonCode Corp.).

### Body growth

Specimens sampled in all years (2004, 2006, and 2013) were used in a comparison of body growth (PAFL length at age) for each otolith type (Table 1). Using the following von Bertalanffy formula [36]:

$$L=L_{inf}\left(1-e^{-K\left(age-to\right)}\right),$$ (4)

estimates of length at age and 95% asymptotic confidence limits were calculated using JMP 12 [37]. Curves were compared to one another with a likelihood ratio test using the R package “fishmethods”.

## Results

### Samples

**Fig 3:**
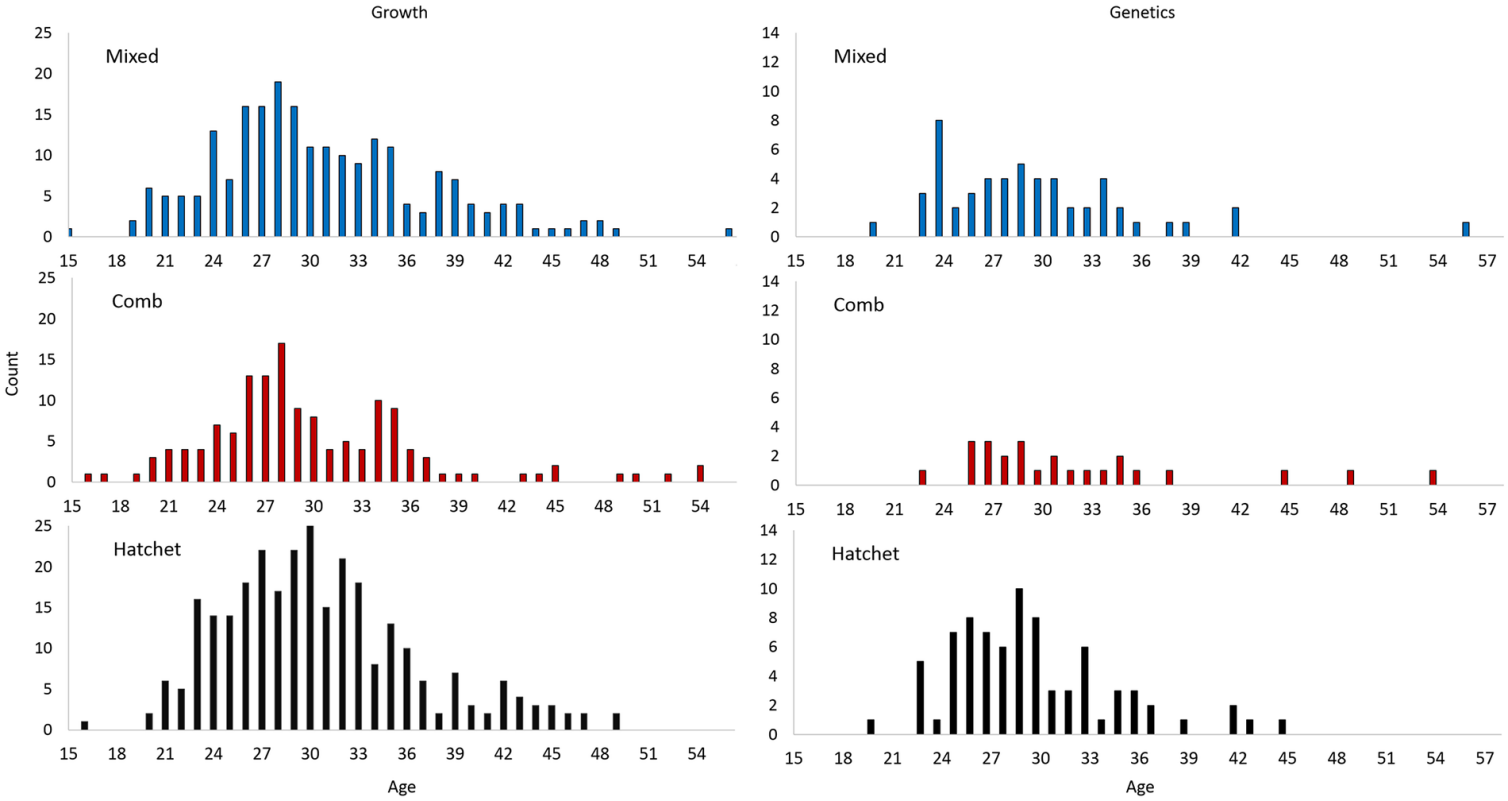
Frequency of giant grenadier by age used in analyses of body growth (growth) and genetics, for mixed-, comb-, and hatchet-shaped otoliths by age.

**Fig 4:**
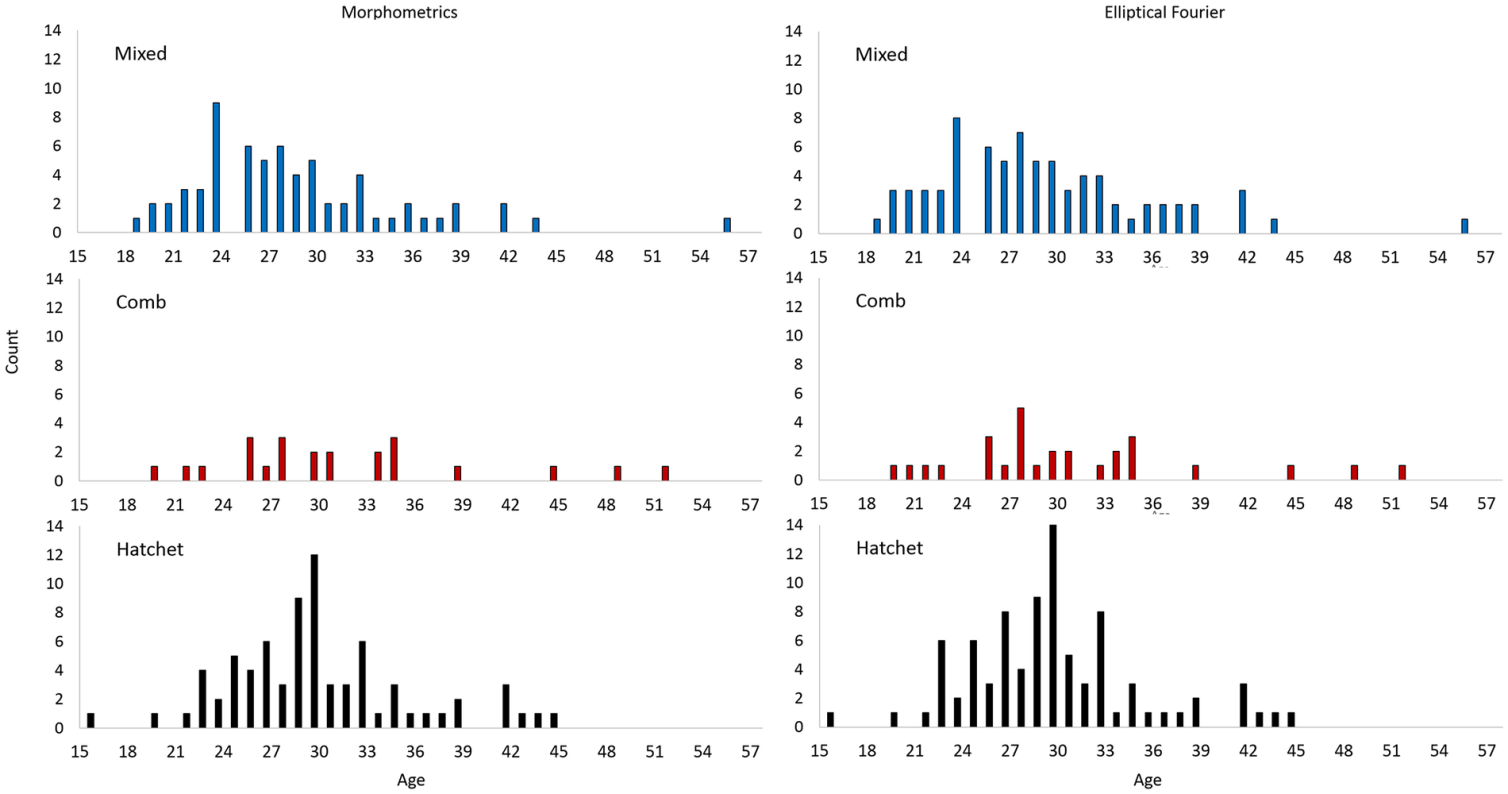
Frequency of giant grenadier by age used in analyses of otolith morphometrics and elliptical Fourier analyses for mixed-, comb-, and hatchet-shaped otoliths by age.

Fish with all three shapes of otoliths were found in all sampling areas in 2004, 2006, and 2013 and a wide age range was sampled for each shape (Tables 1 and 2). A subsample of fish sampled in 2013 were used in genetic, morphometric, and Fourier shape analyses and these subsamples also included a wide age range (Figs 3 and 4). The age range of fish sampled in 2013 and used in the elliptical Fourier analysis was 20–52 years (N = 27) for comb-shaped otoliths was, was 16–45 years (N = 87) for hatchet-shaped, and was 19–56 years (N = 79) for mixed-shaped (Table 2, Fig 4). Samples from 2004, 2006, and 2013 that included fish lengths and ages were used for the growth analysis (Fig 3). The proportions of fish sampled with each otolith shape were very similar in both years in both areas within the Gulf of Alaska (eastern Gulf of Alaska in 2004 and central Gulf of Alaska in 2006) (Table 3).

**Table 3:** Proportion of giant grenadier and total number randomly sampled (in parentheses) in 2004 in the eastern Gulf of Alaska (GOA) and in 2006 in the central GOA by otolith shape.

	Hatchet	Comb	Mixed
eastern GOA	0.44 (72)	0.29 (58)	0.27 (54)
central GOA	0.43 (86)	0.24 (39)	0.32 (54)

### Otolith morphometrics

**Fig 5:**
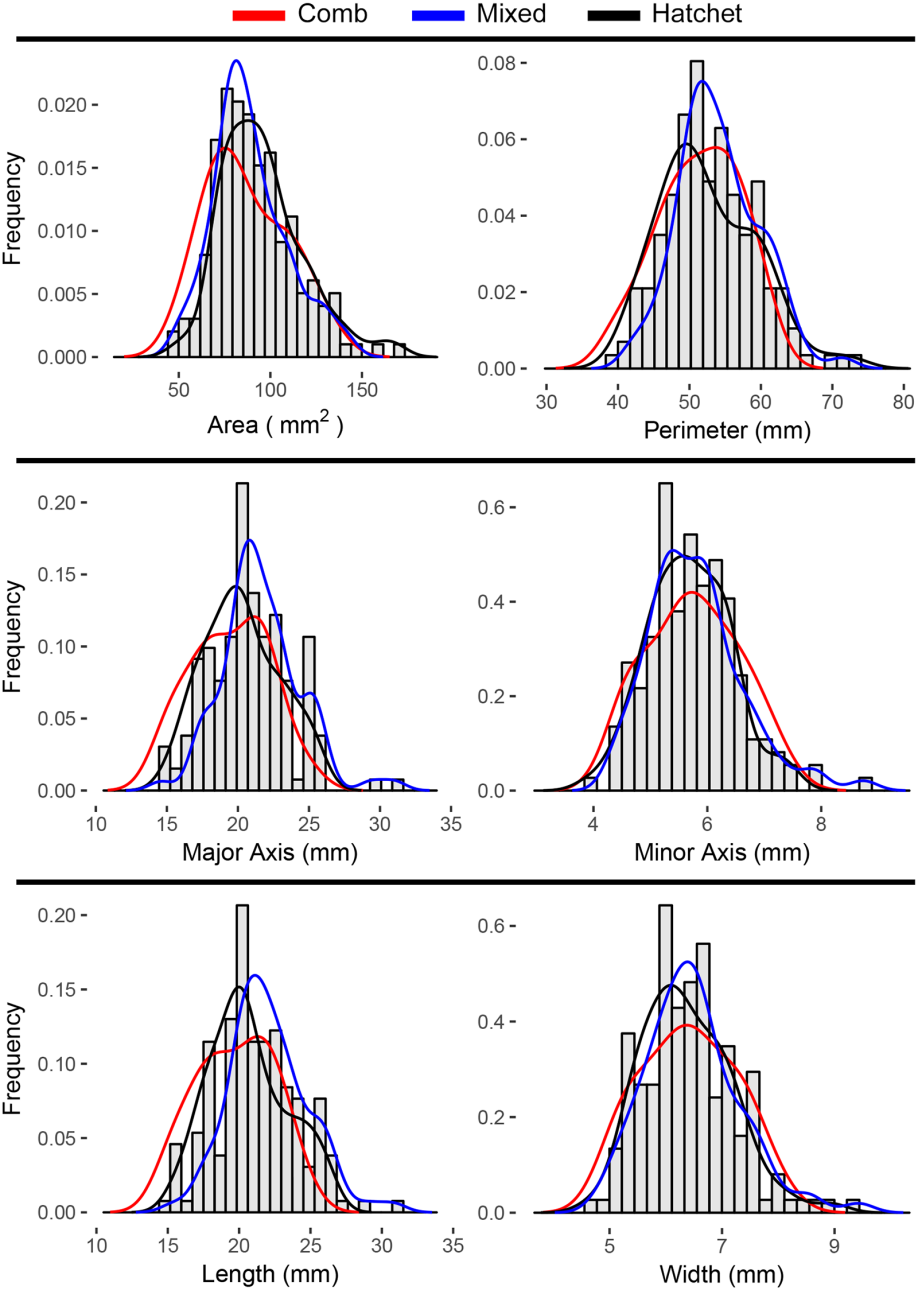
Histograms displaying the frequency for six morphometric measurements from giant grenadier for each shape: Hatchet, comb, and mixed.

Morphometric measurements were taken from 164 specimens. Comb-shaped otoliths had the lowest sample size (Fig 4). The three shapes were not reflected in the otolith morphometrics. That is, there were not multiple modes in the distributions of each measurement (Fig 5). Although morphometrics were not useful for categorizing shapes, in linear models that accounted for fish age and length (one model for each morphometric measurement), otolith Feret length was significantly different among the three shape groups (Table 4). Within a reduced model that excluded interaction terms, because they were not significant, a Tukey-Kramer HSD pairwise test showed that hatchet-shaped otoliths were longer than comb-shaped otoliths (Table 4). There were no other significant differences for any other morphometrics. A PCA based on the morphometric measurements failed to demonstrate a distinct difference between the observed shapes in the grenadier otoliths.

**Table 4:** Tukey-Kramer HSD pairwise comparisons of length for comb-, hatchet-, and mixed-shaped otoliths within a general linear model where the explanatory variables were fish length, age, and otolith shape.

Response	Shape	LS Means	CI	Mean	Sig. difference
Otolith Feret length	comb	20.0	19.2–20.9	19.8	*
	hatchet	21.6	21.1–22.0	21.9	*
	mixed	21.0	20.5–21.5	20.7	

The hatchet- and comb-shaped otoliths were significantly different at ɑ = 0.05, denoted by an asterisk (*). Least squares means (LS Means; mean adjusted for covariates in the model), the 95% confidence interval around the LS mean, and the arithmetic mean (Mean) are reported for each shape.

### Elliptical Fourier shape analyses

**Fig 6:**
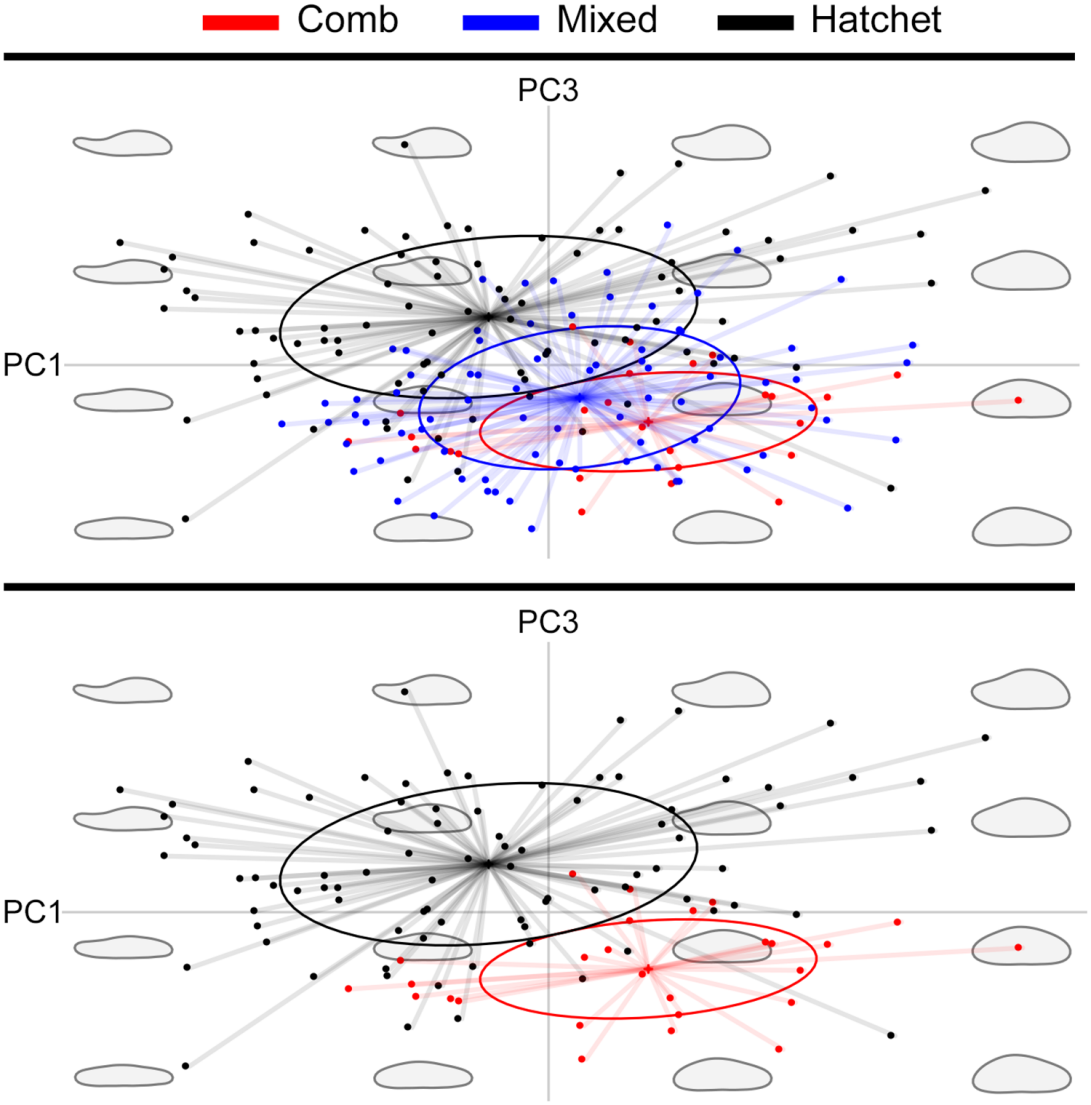
Principal component analysis (PCA) of elliptical Fourier analysis harmonic descriptors for three otolith shapes (comb, hatchet, and mixed) from female giant grenadier. Shapes represent reconstructed outlines based on a range of Fourier descriptors. Ellipses are 90% confidence intervals of the mean shape. Vertical and horizontal lines intersect where PC values are both 0.

**Fig 7:**
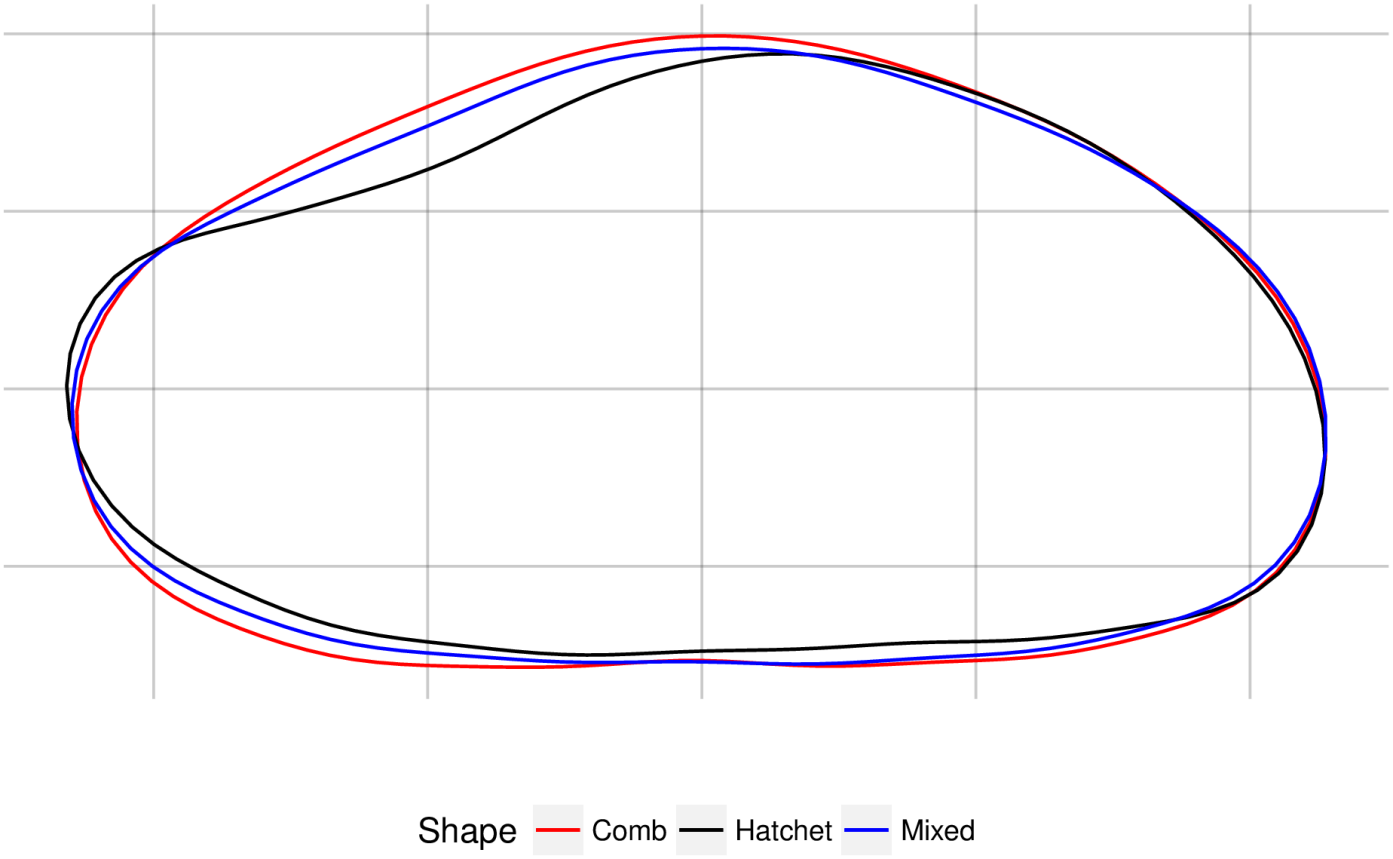
Mean shapes for comb-, mixed-, and hatchet-shaped otoliths for female giant grenadier.

The elliptical Fourier analyses of the smoothed otolith outlines were capable of describing otolith shape using 12 elliptical harmonics, totaling 45 individual descriptors, after three descriptors were removed for adjusting for size and orientation (S1 File). These described 99.8% of the variation in shape. In a MANOVA of the elliptical Fourier analyses descriptors, there was a significant difference among shapes (*F* = 3.022, *p*<0.001). The PCA conducted on these descriptors demonstrated that there was some visual separation between the three otolith shapes in this analysis (Fig 6). A comparison of PC1 to PC3 scores provided the most effective separation of shapes (S2 File). The combination captured the narrow shape on one end of the hatchet-shaped otoliths and the rounded shape of the comb-shaped otoliths, as seen in the reconstructed outlines in Fig 6, which were based on hypothetical Fourier descriptors. In addition, the mean shapes demonstrate that the hatchet shape had a more narrow end than the other two shapes (Fig 7). In the comparison of PC1 and PC3 scores, the 90% confidence region (CR) of the mean shape for comb and hatchet did not overlap, but the 90% CR of the mixed shape intercepted the CR of both the comb and hatchet shapes (Fig 6). The mixed-shaped otoliths overlapped the scores of both other shapes and so there was no clear differentiation between the mixed-shaped otoliths and the other two shapes (Fig 6). In the scatterplot of PC1 and PC2 there was overlap between all three groups (not shown).

**Fig 8:**
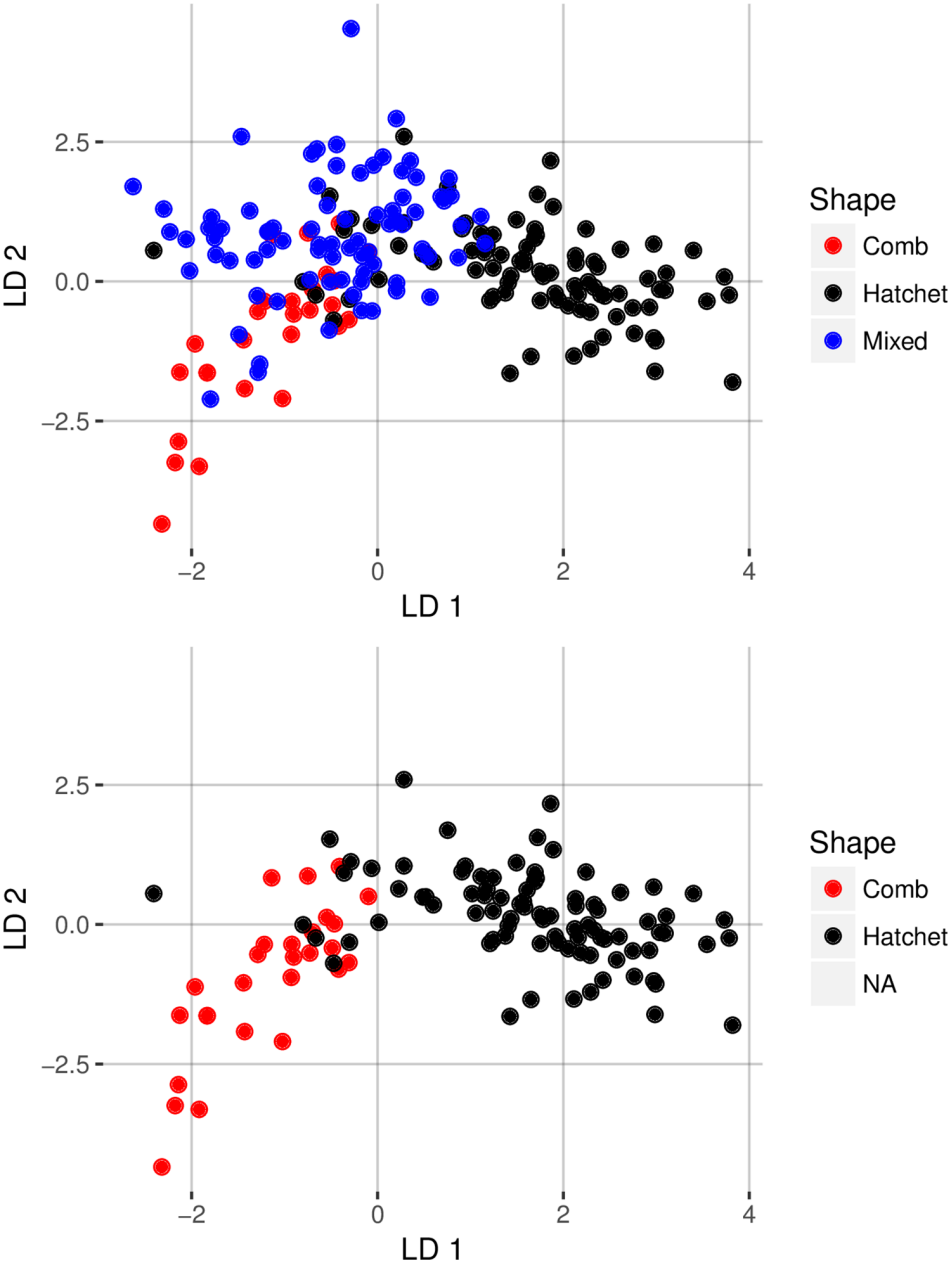
Linear discriminant analysis of elliptical Fourier analysis harmonic descriptors for three otolith shapes (comb, hatchet, and mixed) from giant grenadier. Both plots include the same data except that data from otoliths with a mixed shape are absent from the bottom panel.

Linear discriminant function analysis showed mixed results when attempting to classify otoliths into a shape category (S3 and S4 Files). Two functions were calculated, with the first discriminant weighted at 80.5% and the second at 19.5%. This method specifically emphasizes the maximum variation between groups. Hatchet- and comb-shaped otoliths were almost completely distinct, but the mixed category overlapped both other groups, more so with comb-shaped otoliths (Fig 8). Overall, in the linear discriminate function analysis, 59% of the otolith shape predictions matched the visual classifications (Table 5). Comb-shaped and mixed-shaped otoliths were the most difficult, with 48% and 51% classified correctly, respectively. Hatchet-shaped otoliths were classified correctly 69% of the time. Confusion of comb- and mixed-shaped otoliths caused the majority of misclassifications. For example, more than half (52%) of the otoliths called comb-shaped with visual identification were classified as mixed-shaped with the linear discriminant analysis.

**Table 5:** Classification of otoliths to a shape (either comb, mixed, or hatchet) using a linear discriminant analysis (LDA), compared to shapes identified visually by an experienced age reader (Visual ID). The sample size is the number of samples identified as each shape using visual ID. The number and % under LDA are the sample size and % of the total sample that were identified as each shape using a leave-one-out jack-knife cross-validation. Cells in grey indicate that the visual ID and the LDA were in agreement.

Visual ID		LDA		
Shape	Sample size	Comb	Mixed	Hatchet
Comb	27	13 (48%)	14 (52%)	0 (0%)
Mixed	79	23 (29%)	40 (51%)	16 (20%)
Hatchet	87	7 (8%)	20 (23%)	60 (69%)

### Genetic analysis

Of the 349 individuals sequenced, 172 (50.4%) provided sufficient sequence data (Fig 3). A 535bp segment of the COI gene was analyzed for these 172 individuals. Comparison of COI sequence data failed to separate samples by otolith shape type and sequence variation among all samples was extremely low. Only three samples showed variability from the consensus sequence, and those differed at only a single base each.

### Body growth

**Fig 9:**
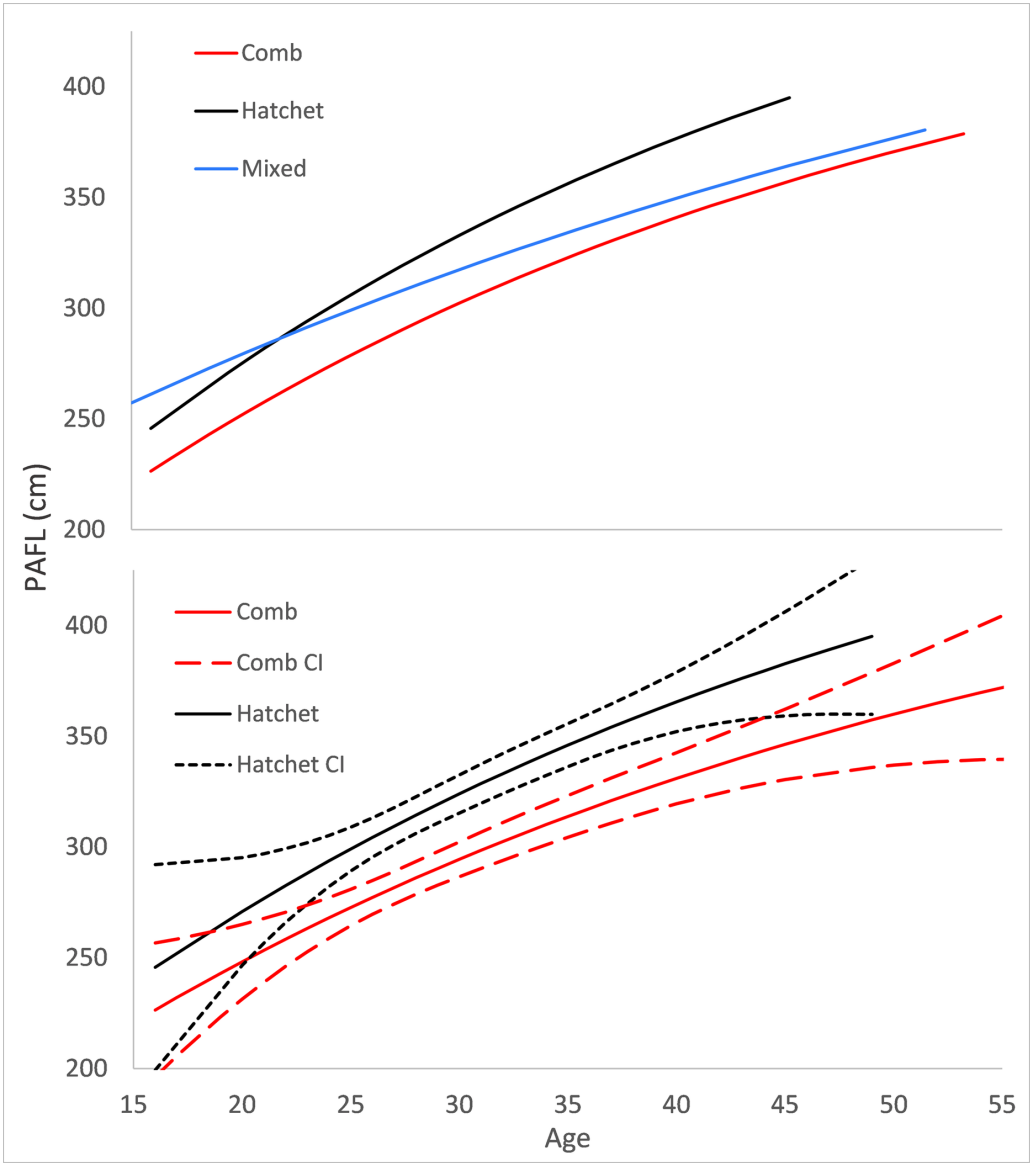
Pre anal fin length-at-age (body growth) for giant grenadier with either comb-, hatchet-, or mixed-shaped otoliths. In the bottom panel the estimated growth curves and 95% asymptotic confidence intervals (CI) are presented for hatchet and comb-shaped otoliths. The majority of samples are between ages 20 and 40. Each growth curve encompasses the age range of each otolith shape.

Overall, fish with a comb-shaped otoliths were not as large at age as those with hatchet-shaped otoliths (χ^2^ = 25.73, p < 0.00) (Fig 9; Table 6). The estimated length of a 45-year old fish with a hatchet-shaped otolith was 11% longer than a fish with a comb-shaped otolith (38.3 vs. 34.6 cm PAFL) (Fig 9). There were few fish over 45 years old sampled, but there were very large differences in the observed (not estimated) size-at-age for these older fish. For example, the oldest fish sampled with a comb-shaped otolith was 58 years old and was 46 cm PAFL and the oldest fish sampled with a hatchet-shaped otolith was 49 years old and was 60 cm PAFL. The growth curve of fish with mixed-shaped otoltihs was not significantly different from other curves (Fig 9). Although not significantly different, the growth curve for the fish with mixed-shaped otoliths was more flat than those for the other two shapes (Fig 9, Table 6). Comb-shaped otoliths had lower sample sizes than other shapes. However, the confidence intervals of the growth curve were still similar in width to the confidence intervals for fish with hatched-shaped otoliths.

**Table 6:** Length-at-age formula parameters for giant grenadier for each otolith shape. L_inf_ is pre anal fin length in millimeters, t0 is age in years, and *N* is the sample size.

	Hatchet	Comb	Mixed
L_inf_	514	469	527
K	-2.46 x 10^−2^	-2.35 x 10^−2^	-1.49 x 10^−2^
t0	-10.40	-12.06	-30.02
*N*	265	129	193

## Discussion

We found that female giant grenadier otoliths had variability in shape among individuals that was detectible both with elliptical Fourier analyses and with the naked eye (Fig 2). Visually, the otoliths were divided into three shape categories (comb, mixed, and hatchet) by an experienced age reader. The two more extreme shapes, comb and hatchet, were more discernable visually and more evident from one another in the principal components scores derived from the elliptical Fourier analyses descriptors, as the comb shape was more rounded than the hatchet shape and the hatchet shape included a long, narrow end. Because of the prominent feature of the narrow end on hatchet-shaped otoliths, the visual and mathematical classification of hatchet-shaped otoliths matched 69% of the time. The mixed shape had characteristics of the comb and hatchet shapes and was difficult to distinguish from the other shapes, particularly from the comb shape. This may partially be an artifact of sample size (there were fewer comb-shaped otoliths analyzed than the other two shapes). Fish with the three otolith shapes were not genetically different. However, because stocks can be identified by a number of parameters, including genetic, behavioral, morphological, and life history strategies, the absence of genetic differences does not necessarily indicate that there is a lack of stock structure in giant grenadier.

Like the observable differences between hatchet- and comb-shaped otoliths, body growth rates of fish with hatchet- and comb-shaped otoliths were distinctive. Fish with comb-shaped otoliths were smaller at age than fish with hatchet-shaped otoliths. The growth of fish with mixed-shaped otoliths overlapped the growth curves of fish with comb- and hatchet-shaped otoliths, akin to the overlap in otolith shapes. Distinctive growth rates have been linked to differences in otolith shape in multiple species (blue whiting (*Micromesistius poutassou*) [4], king mackerel (*Scomberomorus cavalla*) [6], and Atlantic cod (*Gadus morhua*) [3, 16]). In a laboratory study of otolith shape, where larval and juvenile Atlantic cod on a higher food ration had a wider otolith with more otolith lobes [3]). The results from that study indicated that at least one component of otolith shape variability is a phenotypic response to feeding level and growth. In the wild, otolith shape variation is often coupled with differing geography for at least a portion of the year, such as stocks with variation in spawning migrations or feeding grounds [3, 4, 7, 8, 9, 12], which may translate to differences in food availability, environmental conditions, and growth rates. Because data on the larval and juveniles stages (ages <15 years) of giant grenadier and the movement of adults are lacking, we cannot relate their behavior, life history, or habitat to the variation observed in otolith shape or growth, although much of the literature points to environmental factors as the major influence on otolith shape [3, 4, 8, 14].

We did not find any genetic variation in giant grenadier. However, the COI locus is used for identifying species level differences and not fine-scale stock structure. We chose this technique because we hypothesized that the extreme variation in otolith shape could be indicative of a different species. It is possible that more fine-scale genetic differences exist in giant grenadier and could be identified using other genetic techniques. Genetic differences are often subtle or absent in marine species, even when phenotypic differences in otolith shape are measurable [3, 4, 14]. In the literature, otolith shape within a species has only beenlinked to a genetic component in few instances [15], but has been strongly linked to life history and environmental differences coupled with little to no genetic differences [4, 14]. Therefore, even without an observed genetic component for otolith shape, some level of stock structure may be associated with otolith shape.

The elliptical Fourier and linear discriminate analyses illustrated that there were quantifiable differences between the shapes visually identified as comb and hatchet, but the mixed-shape overlapped both of these shapes. It is possible that for giant grenadier the average shape is what we described as a mixed shape and the comb and hatchet shapes are the extremities of a broad spectrum, implying a single stock. Alternatively, the shapes could be associated with specific life histories, and potentially indicate stock structure. Some overlap in shapes is not unusual, even when there is known stock structure. For example, when otolith shape was used for stock identification there was low classification success (0–44%) for Icelandic cod stocks of Atlantic cod [8] and high success (89–90%) for northeast arctic Atlantic cod [3]. The rate of success of stock differentiation depends on the degree of difference between the shapes. The spectrum of shapes we observed could be due to fish co-occurring in the same environment during some early life stages and not during others, which is the case for southern blue whiting (*Micromesistius australis*) [12]. This co-occurrence during some life stages could be experienced by individuals and not determined by stock-specific ontogenetic or seasonal movements. The mechanisms driving shape diversity in giant grenadier remain unknown because there is very little information on giant grenadier habitat use or movement at any life stage, and females that were sampled in the current study had high variability in otolith shape at the same capture sites.

Our data included information on capture depth but there were not enough samples to conduct an analysis on the effect of depth on otolith shape. There is almost nothing known about habitat use before giant grenadier settle to the benthic environment on the continental slope. Studies of behavior or movement are not possible because grenadier die from barotrauma when brought to the surface. However, catch and size data are available from surveys. In a deepwater longline survey the western Gulf of Alaska during the summer, body size and sex ratio changed as sampling descended deeper than 1,000 m. At depths >1,000 m females were on average 69% heavier than at depths < 1,000 m [24]. This demonstrates that female grenadier must move vertically with age or size. Also, the proportion of males at depths <1,000 m was on average 3% and increased dramatically at depths from 1,000–1,600 m to 12–42%, depending on the sampling site. Because males are found in deeper water, at least during the summer months, there must be movement associated with spawning for at least one sex. This movement could be related to the otolith shape. However, it is unknown when movements for spawning occur because females with ovaries at all stages of development were observed in the summer and so their spawning season is likely protracted [21]. Behavioral observations of giant grenadier from video taken from submersibles can be used to provide information on the ability of giant grenadier to undertake migrations. These videos show that giant grenadier have total spine flexion, much like eels (order Anguilliformes), and are often observed pointed into the current and moving continually to keep the same position just off-bottom (R. Stone, Alaska Fisheries Science Center, National Oceanic and Atmospheric Administration, pers. comm.). They may sustain this position for feeding on prey drifting toward them in the current. These observations demonstrate that giant grenadier have the ability to maintain a slow, steady swimming speed, likely for long duration. This tendency to stay off-bottom and their swimming ability indicate that they are not likely a sedentary species and are capable of movements associated with ontogeny or spawning.

There are many directions for future work to investigate the mechanisms driving the otolith and body shape differences we observed. We did not have the sample size to examine growth and shape by depth. A larger number of samples covering the depth range in Alaska (all areas on the continental slope from 400 m down to, a minimum, of 1,600 m [24]) would allow for an analysis of the effect of depth. We did not include males for this study because there were almost none caught at the depths sampled. A study including males would allow for an examination of otolith shape and body growth on a species level. We assumed that samples were independently collect from one population within our study area because all shapes were found in each area and the proportions of shapes, in areas where samples were collected randomly, were very similar. This could be tested further by randomly sampling fish throughout their range for a measurement of the proportion of each shape in each area, including areas off the west coast of the United States, British Columbia, Canada, and Russia. A propensity for a shape and/or and life stage in some areas or depths could indicate that there are otolith shapes associated with habitat or life history. In the future, surveys of pelagic waters may be fruitful for identifying where giant grenadier <15 years old reside and at what life stage the otolith shape begins to diverge.

## Supporting information

S1 File Elliptical Fourier descriptors. (CSV)

S2 File Elliptical Fourier principal component analysis values. (CSV)

S3 File Leave-one-out cross-validation probabilities for the linear discriminate analysis. (CSV)

S4 File Linear discriminant analysis values. (CSV)
